# Role of CARD9 in inflammatory signal pathway of peritoneal macrophages in severe acute pancreatitis

**DOI:** 10.1111/jcmm.15559

**Published:** 2020-08-12

**Authors:** Jing Wang, Jun Tian, Yang‐huan He, Zhi‐wen Yang, Lin Wang, Yue‐xing Lai, Ping Xu

**Affiliations:** ^1^ Songjiang Hospital Affiliated to Shanghai Jiaotong University School of Medicine (Preparatory Stage) Shanghai China; ^2^ Shanghai Songjiang Clinical Medical College of Nanjing Medical University Nanjing China; ^3^ Jinshan Hospital of Fudan University Shanghai China

**Keywords:** caspase recruitment domain‐containing protein 9, Dectin‐1, macrophages, severe acute pancreatitis, toll‐like receptor 4

## Abstract

Previous studies revealed that caspase recruitment domain protein 9 (CARD9) was involved in severe acute pancreatitis (SAP) inflammation and that interfering with its expression in vivo could inhibit inflammation. However, the specific mechanism is unknown. This study aimed to discover the related signal pathways of CARD9 in macrophages. SiRNA interference technology was used in vivo and in vitro to detect CARD9‐related signal pathways in peritoneal macrophages. Furthermore, Toll‐like receptor 4 (TLR4) and membrane‐associated C‐type lectin‐1 (Dectin‐1) pathways in macrophages were activated specially to looking for the upstream signal path of CARD9. Results showed up‐regulation of CARD9 expression in peritoneal macrophages of SAP rats (*P* < .05). CARD9 siRNA alleviated inflammatory cytokines, and inhibited the phosphorylation of NF‐κB and p38MAPK in peritoneal macrophages in vivo or in vitro. Meanwhile, CARD9 siRNA reduced the concentration of CARD9 and Bcl10 in peritoneal macrophages, and TLR4 and Dectin‐1 took part in CARD9 signal pathways in macrophages. In conclusion, there is an inflammation signal pathway comprised of TLR4/Dectin‐1‐CARD9‐NF‐κB/p38MAPK activated in macrophages in SAP. Blockade of CARD9 expression in macrophages can effectively alleviate SAP inflammation.

## 1 INTRODUCTION

The pathogenesis of severe acute pancreatitis (SAP) is complex. Over‐product of pro‐inflammatory cytokines plays a key role in the rapid spread and amplification of local inflammatory responses in the pancreas to the whole body.^1^ Macrophage activation is very important in the early events of SAP.^1^, ^2^ Many kinds of stimulation activate macrophages, leading to the release of inflammatory cytokines and activate neutrophils, vascular endothelial cells and macrophages themselves, aggravating the systemic inflammatory response, and ultimately leading to multiple organ failure, even death.^3^

Caspase recruitment domain protein 9 (CARD9) is highly expressed in macrophages.^4^ It is an important adaptor protein, which can efficiently integrate the recognition signals of various innate immune receptors, and regulate the intracellular signal transmission to play an important role in the innate immunity.^5^ Recently, articles about CARD9 have mainly focused on infectious inflammation,^6^, ^7^, ^8^, ^9^ but there have been no researches reporting on the relationship between CARD9 and SAP before our studies.

Previous studies by our group revealed that CARD9 in peripheral blood monocytes of patients with SAP increased significantly.^10^ Furthermore, in our animal experiments, the mRNA and protein level of CARD9 in pancreatic tissue is higher in SAP rats, while adenovirus interfered with the expression of CARD9, which effectively alleviated the inflammatory response of pancreatic tissue.^11^ Therefore, these previous studies demonstrated that CARD9 was involved in SAP inflammation and that interfering with its expression could inhibit inflammation. However, some problems still remain unsolved. This study aimed to discover the related signal pathway of CARD9 in macrophages in vivo and in vitro via siRNA interference technology, and to explore the possible molecular mechanisms of CARD9 in SAP.

## 2 MATERIALS AND METHODS

### 2.1 Animal model of SAP

Healthy male Sprague‐Dawley rats weighing 230‐260 g were used to establish SAP animal model. These rats are provided by Shanghai Jie Si Jie Laboratory Animal Co. Ltd (Animal permit number: SCXK (Hu) 20130006). Standard deviation (SD) rats are fed clean grade for one week. 12 hours fasting and free drinking before operation.^11^

### 2.2 Inhibiting CARD9 expression in vivo

Obio Technology Co., Ltd succeeded in designing and synthesizing adenoviral constructs incorporating siRNA against CARD9. The sequence number of CARD9 in GenBank is NM_022303. The target sequences were 5′‐GCTTTCAGGACAAAGATAA‐3′ (CARD9) and 5′‐TTCTCCGAACGTGTCACGT‐3′ (control). 1 × 10^9^ plaque‐forming units (PFUs) of CARD9 siRNA were injected into caudal vein.

Standard deviation rats were divided into four groups: Control group (Control, n = 6), rats underwent a sham operation with nothing infused; SAP group (SAP, n = 18), 5.0% sodium taurocholate was retrograde perfusion into the pancreatic and biliary duct, the dosage of sodium taurocholate is 1 mL/kg bodyweight; siCARD9 + SAP group (siCARD9, n = 18), 1 × 10^9^ PFUs of CARD9 siRNA (dissolved in 200 μL PBS) were injected into caudal vein, and SAP model was induced in the same manner as the SAP group 48 hours later; sicontrol + SAP group (sicontrol, n = 18), the same manner as the (siCARD9 + SAP) group, control siRNA instead of CARD9 siRNA. Following the treatments, rats were fast and free to drink water. Peripheral blood, ascitic fluid and pancreatic tissue were collected 3, 6 and 12 hours after modelling. Experimental animal part was approved by the Ethical and Research Committee of Shanghai Jiao Tong University, and was designed and implemented in strict accordance with ‘the guidelines for the care and use of experimental animals in research’.

### 2.3 Isolation and culture of peritoneal macrophages

At 3, 6 and 12 hours after the establishment of the model, peritoneal effusions from the SAP rat groups were extracted under an aseptic environment, and peritoneal lavage was performed with 25 mL precooled PBS two consecutive times. The control group rats were given peritoneal lavage directly. The peritoneal lavage fluid and ascites were mixed evenly and the peritoneal macrophages were separated by density gradient centrifugation at 4°C, centrifuged at 500 × g for 15 minutes, then at 120 × g for 3 minutes. Mixed precipitated cells were mixed with DMEM complete medium (10% foetal bovine serum, penicillin and streptomycin) and evenly distributed in 12‐well plates. The plates were incubated under 5% CO_2_ at 37°C. After 3 hours, the macrophages were washed twice with PBS to remove unattached cells. After 24 hours of incubation, RNA and protein were collected from cells lysed in Trizol and RIPA for real‐time PCR and Western blot detection, respectively.

### 2.4 Inhibiting CARD9 expression in vitro

The purified peritoneal macrophages were cultured for 24 hours and then mixed with adenoviruses expressing siRNA against CARD9. The concentration of the original viral solution was 1 × 10^11^ PFU/mL. The original solution was diluted according to the amount of adenovirus interference with multiples of infection (MOIs) of 1:100, 1:200 and 1:500. The diluted adenoviruses were then mixed in 200 μL medium without foetal bovine serum and added to each well. Peritoneal macrophages and adenoviruses were cultured together for 3 hours, then the supernatant was removed and added medium containing foetal bovine serum for 24 hours. Adenovirus transfection and cell viability were observed by inverted fluorescence microscopy to determine the optimal MOI for subsequent experiments.

Rats were randomly divided into two groups: Normal group (n = 6), rats without any treatment, and SAP model group (n = 36), using the previously described method for establishing SAP. Isolation and purification of peritoneal macrophages were performed as described above 6 hours after establishment of the model. After 24 hours incubation, peritoneal macrophages from the SAP group were randomly divided into three groups: SAP group (SAP), cells incubated with PBS; siCARD9 + SAP group (siCARD9), cells incubated with 5 × 10^8^ PFU of CARD9 siRNA; sicontrol + SAP group (sicontrol), cells incubated with 5 × 10^8^ PFU of control siRNA. Peritoneal macrophages from normal rats were incubated in culture medium and were used as the control group (Blank). The cells were cultured in 12 well plates with 1 mL cell suspension per well and 5 × 10^5^ cells per mL. After 24 hours of culture, cells were collected and centrifuged to obtain cell lysates and culture supernatant, which were frozen for subsequent detection.

### 2.5 Polyinosine andβ‐glucan stimulation of primary peritoneal macrophages

The rats were randomly divided into two groups: Normal group (n = 18), rats without any treatment, and the SAP model group (n = 30), using the previously described method for establishing SAP. Isolation and purification of peritoneal macrophages were performed 6 hours after the establishment of the SAP model. After 24 hours incubation, peritoneal macrophages from the normal group were randomly divided into three groups: *Control group (Blank)*, cells incubated in culture medium without any siRNA or drugs; *polyinosine group (Poly)*: cells incubated with a final concentration of 20 μg/mL polyinosine (Sigma‐Aldrich, St. Louis, MO, USA); and *β‐glucan group (β‐glucan)*, cells incubated with a final concentration of 100 μg/mL β‐glucan (Sigma‐Aldrich). The peritoneal macrophages from the SAP model group were randomly divided into five groups: *SAP group (SAP)*, cells incubated in culture medium without any drug; *SAP + polyinosine group (SAP + Poly)*, cells incubated same as *polyinosine group*; *SAP + siCARD9 + polyinosine group (SAP + siCARD9 + Poly)*, cells incubated with CARD9 siRNA (5 × 10^8^ PFU) 24 hours before treatment with polyinosine; *SAP + β‐glucan group (SAP + β‐glucan)*, cells incubated same as β‐glucan *group*; *SAP + siCARD9 + β‐glucan group (SAP + siCARD9 + β‐glucan)*, cells incubated with CARD9 siRNA (5 × 10^8^ PFU) 24 hours before treatment with β‐glucan. After 24 hours of culture, cells were collected and centrifuged to obtain cell lysates and supernatant, which were frozen for subsequent detection.

### 2.6 Histological examinations

The pancreatic tissue was fixed with 10%formalin and made into wax block, then sectioned and stained with haematoxylin and eosin (H&E). The histological changes of the pancreatic tissue were observed by the pathologist under light microscope, and evaluated from the four aspects of inflammation, oedema, haemorrhage and necrosis. The score of each aspect was from 0 to 4 according to the severity, and the total score was pathological score.^11^

### 2.7 ELISA

The level of tumour necrosis factor‐α (TNF‐α), interleukin‐1β (IL‐1β) and interleukin‐6 (IL‐6) in peritoneal macrophage supernatants were assayed with ELISA kits (eBioscience), according to the manufacturer's instructions.

### 2.8 Real‐time PCR

The total RNA was extracted from 1 × 10^6^ fresh adherent peritoneal macrophages by Trizol one‐step method in strict accordance with the instructions of the reagent (TaKaRa Bio Inc.). The mRNA expression of Toll‐like receptor 4 (TLR4), membrane‐associated C‐type lectin‐1 (Dectin‐1), CARD9, TNF‐α, IL‐1β and IL‐6 mRNA in peritoneal macrophages were detected by two‐step PCR reaction procedure. GAPDH was used as internal reference gene for calculation. The gene‐specific primers sequence in Table 1. The amplification conditions was: first, pre denaturation: 95°C for 10 minutes; second, PCR reaction: 39 cycles of 95°C for 15 seconds, 60°C for 45 seconds; the third step is decomposition stage: 95° 15 seconds + 60° 1 minute + 95° 15 seconds, collecting the fluorescence of each PCR extension period. 2^−^ *^△△C^* ^t^ was used to express the relative level of related genes.

**Table 1:** The gene‐specific primers

	Sense	Antisense
*CARD9*	5′‐ATTGACCCCTCACGAATCAC‐3′	5′‐AGGAGCACACCCACTTTCC‐3
*TLR4*	5′‐CCACAAGAGCCGGAAAGTTA‐3′	5′‐GAAACTGCCATGTCTGAGCA‐3′
*Dectin‐1*	5′‐GACTTCGGCACTCGAAACAT‐3′	5′‐TCCTAAAGCTACCGCAATGG‐3′
*TNF‐α*	5′‐AGATGTGGAACTGGCAGAGG‐3′	5′‐CGAGCAGGAATGAGAAGAGG‐3′
*IL‐1β*	5′‐CAGGAAGGCAGTGTCACTCA‐3′	5′‐AAAGAAGGTGCTTGGGTCCT‐3′
*IL‐6*	5′‐AGTTGCCTTCTTGGGACTGA‐3′	5′‐ACTGGTCTGTTGTGGGTGGT‐3′
*GAPDH*	5′‐GTCGGTGTGAACGGATTTG‐3′	5′‐TCCCATTCTCAGCCTTGAC‐3′

### 2.9 Western blot

Protein samples were prepared using PRO‐PREP protein extraction solution (Beyotime Institute of Biotechnology) for total fractions according to the manufacturer's instructions.^12^ Total protein was separated by sodium dodecyl sulphate‐polyacrylamide gel electrophoresis (SDS‐PAGE) and transferred to polyvinylidene fluoride membranes (Millipore). The membranes were blocked with 5% non‐fat milk for 1 hour, incubated overnight at 4°C with antiserum against CARD9, NF‐κBp65, p38MAPK, TLR4, Dectin‐1 (1:1000 respectively, Abcam), p‐NF‐κBp65, p‐p38MAPK (1:1000 respectively, CST), and then incubated with the secondary antibodies for 1 hour. Antibodies against β‐actin (1:10 000, Abcam, Cambridge, UK) or GAPDH (1:5000, Abcam) were used as an internal control. Then, chemiluminescence detection was carried out, using ECL Plus kit (Amersham Oxford, UK). Optimus software was used to quantitate Densitometric analyses (Optimus Corp).

### 2.10 Immunoprecipitation

Interaction between CARD9 and B‐cell lymphoma factor 10 (Bcl10) in peritoneal macrophages was detected by immunoprecipitation according to the manufacturer's instructions. Briefly, peritoneal macrophages (2 × 10^6^) from the animal models were lysed in 500 μL cold RIPA buffer. Lysates were pre‐cleared with uncoated Dynabeads (Invitrogen). Anti CARD9 (Santa Cruz, CA, USA) and BCL‐10 antibody (CST) together with protein A/G agarose were used to immunoprecipitate BCL‐10 and CARD9, respectively. Then, proteins were resolved by SDS‐PAGE and analysed by Western blot to detect whether there was interaction between BCL‐10 and CARD9.^13^

### 2.11 Electrophoretic Mobility Shift Assay (EMSA)

EMSA was to detect the transcription activity of NFκB. The nuclear extracts were extracted according to the instructions of the kit (Thermo Scientific) and stored at −80℃. Protein concentration was measured using a BCA protein assay kit (Pierce, Biotechnology) and specific probes were synthesized (Sangon Biotech). EMSA glue (5% non‐denaturing gel) was prepared; the EMSA binding reaction system (5 × gel shift buffer 2 μL + nucleoprotein 1 μL + biotin‐labelled probe 1 μL + nucleus+NFκB 1 μL (Abcam) + free water 4 μL) was prepared and mixed in 200 μL PCR tube, then incubated at 30℃ for 5 min, and then added with 1 μL heparin sulphate (50 mg/mL) for 5 minutes on ice. Add 1 μL 10 × loading buffer, mix well, and immediately sample for electrophoresis and membrane transfer. At the end of crosslinking, chemiluminescence detection is performed immediately. Densitometric analyses were conducted using Optimus software (Optimus Corp) for quantitation.

### 2.12 Statistical analysis

Results are presented as means ± SD. The statistical significance was assessed through a one‐way analysis of variance followed by SNK using SPSS 21.0 software (IBM). All tests were two‐tailed, and differences were considered significant at *P* < .05.

## 3 RESULTS

### 3.1 Up‐regulation of CARD9 expression in macrophages of SAP rats

**Figure 1:**
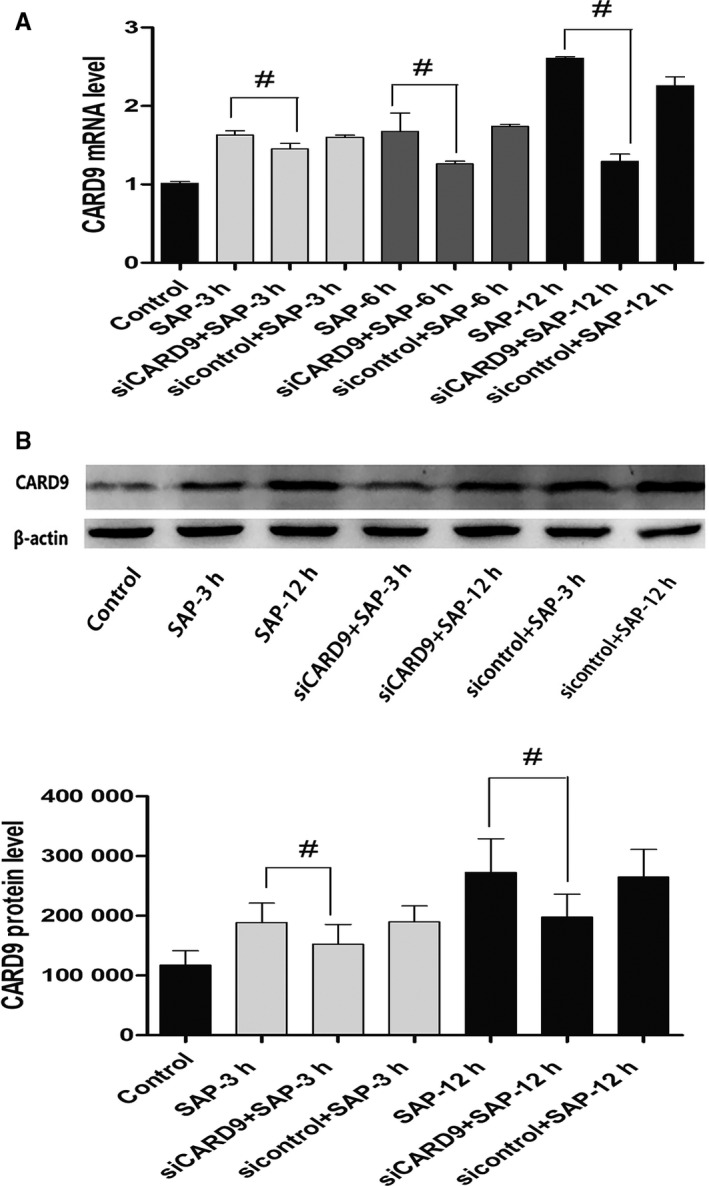
CARD9 expression in peritoneal macrophages of SAP rats. A, mRNA expression of CARD9 in peritoneal macrophages in each group. B, Protein expression of CARD9 in peritoneal macrophages in each group. The molecular weight of the CARD9 bands was 62 kD. The change in protein expression was consistent with changes in gene expression. The siCARD9 group successfully demonstrated interrupted expression of CARD9. #: SAP group vs siCARD9 group, *P* < .05

Compared with the control group, the mRNA level of CARD9 in peritoneal macrophages in the SAP group increased significantly at 3, 6 and 12 hours after induction of SAP, and was highest at 12 hours (*P* < .05) (Figure 1). The expression of CARD9 protein in peritoneal macrophages from SAP rats was also higher than that in control group (*P* < .05) (Figure 1). These data showed that CARD9 participates in the inflammatory responses.

### 3.2 CARD9 siRNA inhibits inflammatory cytokines and the inflammatory signalling pathway in peritoneal macrophages in vivo and in vitro

#### 3.2.1 CARD9 siRNA knockdown in vivo

Adenoviral constructs carrying CARD9 siRNA were injected into the tail vein 48 hours before the SAP model was established in rats, followed by interference efficiency detection. The mRNA and protein expression of CARD9 in the siCard9 group were decreased sharply compared to the SAP group at 3, 6 and 12 hours. Furthermore, siCard9 rats showed a reduction of up to 60% in CARD9 mRNA expression (Figure 1). Control siRNA rats showed no differences in CARD9 expression compared to SAP rats (Figure 1). The results suggested that *CARD9* gene silencing with siRNA by caudal vein injection could significantly inhibit CARD9 expression in peritoneal macrophages.

#### 3.2.2 CARD9 siRNA knockdown in vitro

**Figure 2:**
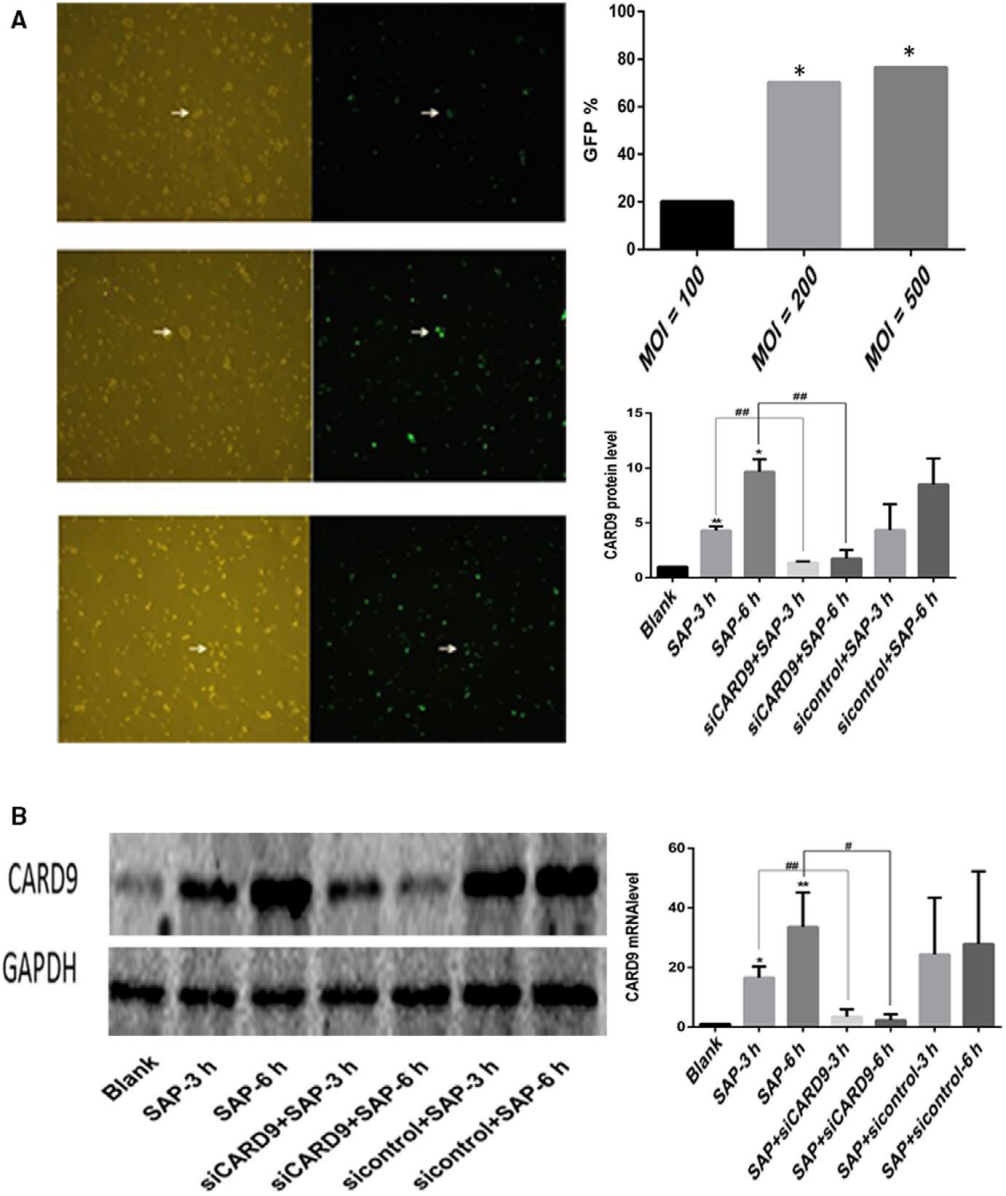
CARD9 siRNA knockdown in vitro. A, Adenovirus transfection of rat peritoneal macrophages in vitro. B, The expression of CARD9 in peritoneal macrophages from each group. The molecular weight of the CARD9 bands was 62 kD. *: SAP group or siCARD9 or sicontrol group vs control group, *P* < .05; #: SAP group vs siCARD9 group, *P* < .05

We use the expression of green fluorescent protein (GFP) to reflect transfection efficiency. The expression of GFP in primary peritoneal macrophages of SAP rats after adenovirus transfection is shown in the Figure 4A. When MOI was 100, 200 and 500, the positive rates of GFP expression were 20.13 ± 2.63%, 70.23 ± 4.66 and 76.54 ± 4.78%, respectively. With the increase of MOI, the percentage of GFP positive cells gradually increased. Statistical analysis showed that there were significant differences in the percentage of GFP positive cells among different MOI virus treatments. Cell counts under high power microscopy showed that when MOI was 500, more cells died. However, when the MOI was 200, the positive rate of GFP expression was high and the cell activity was good. Therefore, MOI 200 was chosen for follow‐up tests. (Figure 2A).

After extraction of peritoneal macrophages from the siCARD9 adenovirus‐treated group and the sicontrol group, the expression of CARD9 mRNA and protein in the siCARD9 group was lower than in the SAP and sicontrol groups. Meanwhile, the expression of CARD9 in SAP and sicontrol groups was higher than in the blank group. Furthermore, the siCARD9 group showed a reduction of up to 60% in *CARD9* mRNA expression (Figure 2B).

#### 3.2.3 CARD9 siRNA alleviates pancreatitis severity in SAP rats and inhibits inflammatory cytokines in peritoneal macrophages in vivo and in vitro

**Figure 3:**
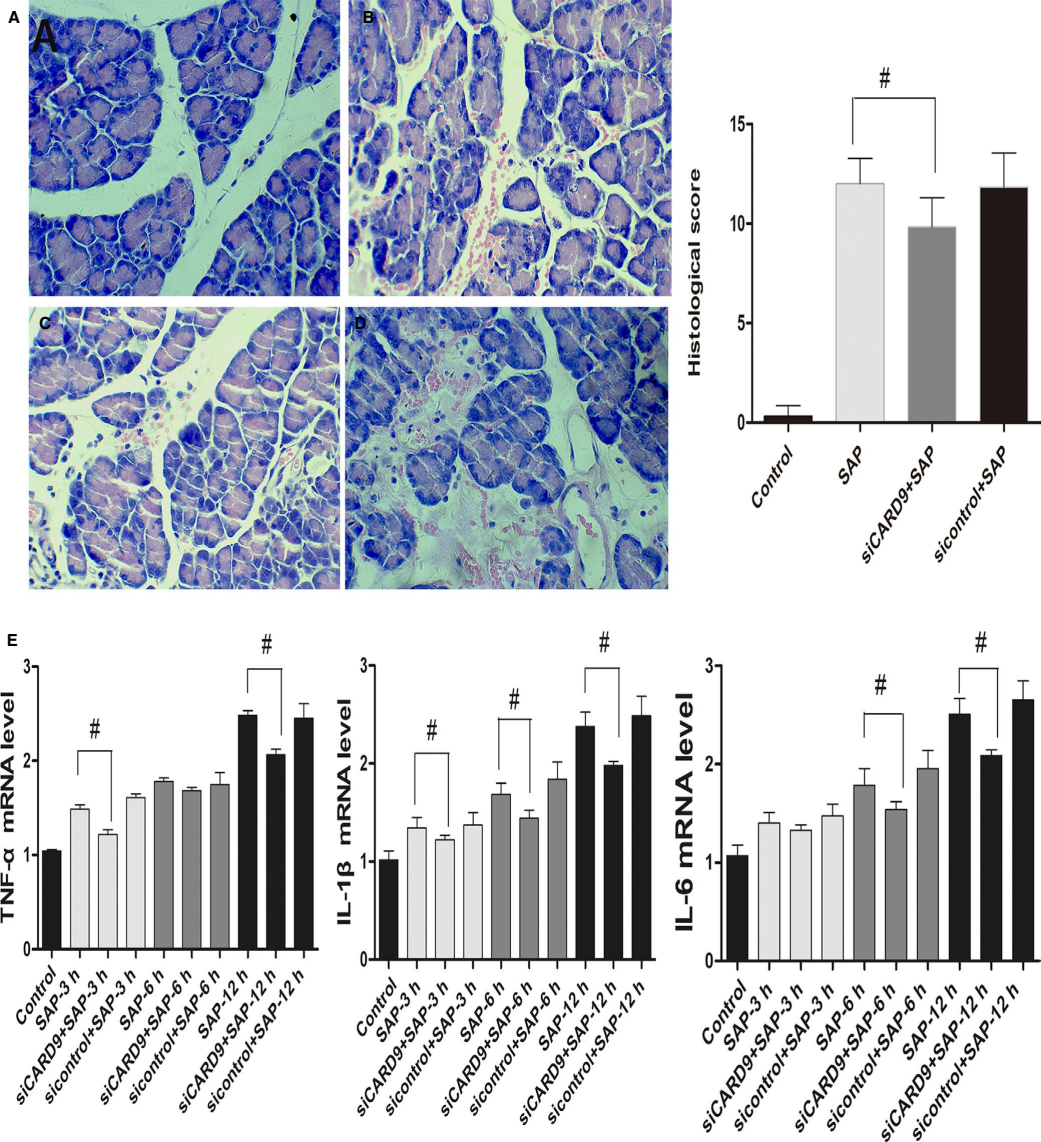
In vivo CARD9 siRNA alleviates pancreatitis severity and inhibits inflammatory cytokines in peritoneal macrophages. A, Histological examination of the pancreas in each group at the 12‐h time point (original magnification, 200×) and pathological scores for pancreatic tissue in each group. a: sham‐operation group; b: SAP group; c: siRNA group; d: sicontrol group. B, The level of TNF‐α, IL‐6 and IL‐1β in each group of peritoneal macrophages from SAP rats. #: SAP group vs siCARD9 group, *P* < .05

In our preliminary work, results showed that compared to the SAP group, *CARD9* gene silencing caused a reduction in ascites volume and obvious amelioration of pancreatic injury.^11^ In this study, as shown in Figure 3A we got the same results in pathological changes and pathological scores of pancreas in (siCARD9 + SAP) group at 6 and 12 hours. It indicates that the moulding is successful and interfering with the expression of CARD9 in peritoneal macrophages in vivo can effectively reduce the inflammatory response of pancreatic tissue.

**Figure 4:**
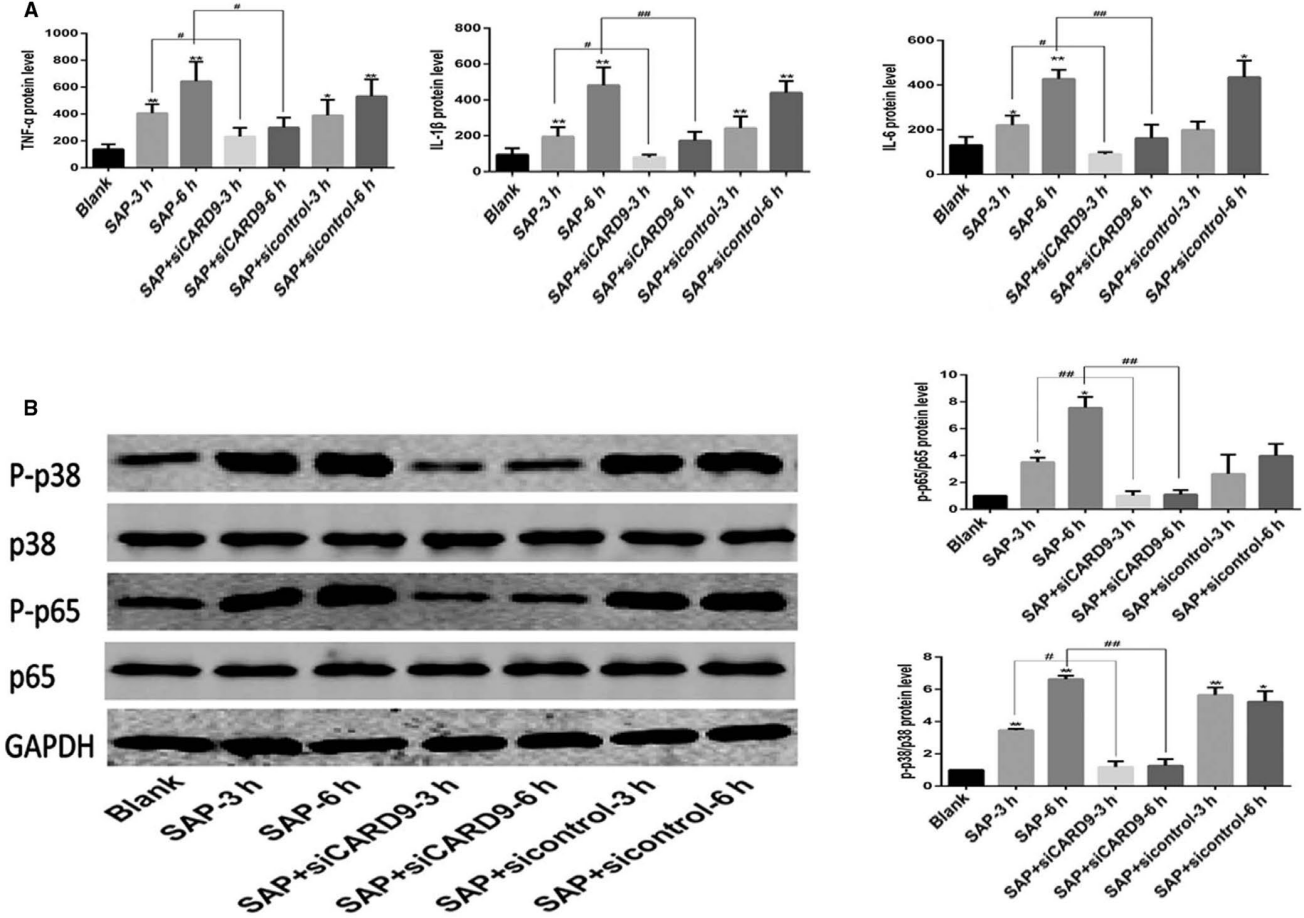
In vitro CARD9 siRNA alleviates Inflammatory reactions in peritoneal macrophages. A, The level of TNF‐α, IL‐1β and IL‐6 in each group of peritoneal macrophages. B, Phosphorylation of NF‐κB and P38 in peritoneal macrophages from each group at the 3‐ and 6‐h time points. The molecular weight of the NF‐κBp65 and P38 bands were 65 kD and 43 kD. *: SAP group or SAP + sicontrol group vs Blank group, *P* < .05; **: SAP group or SAP + sicontrol group vs Blank group, *P* < .01; #: SAP + siCARD9 group vs SAP group, *P* < .05; ##: SAP + siCARD9 group vs SAP group, *P* < .01

TNF‐α, IL‐6 and IL‐1β levels were correlated with the severity of SAP and pancreatic injury. In vivo, the mRNA level of these inflammatory factors in peritoneal macrophages in SAP group was higher than that in control group and in (siCARD9 + SAP) group at 6 and 12 hours. There was no significant difference between the SAP group and sicontrol group (Figure 3B). In vitro, as shown in Figure 4A–C, the levels of TNF‐α, IL‐1β and IL‐6 in cell culture medium from the siCARD9 group were significantly lower than in the SAP and sicontrol groups. Meanwhile, the levels of TNF‐α, IL‐1β and IL‐6 in the latter two groups were significantly higher than in the blank group. There were statistically significant differences in all comparisons (*P* < .05), especially for IL‐1β and IL‐6 at 6 hours (*P* < .01). These data confirming that CARD9 siRNA could reduce the secretion of inflammatory factors in peritoneal macrophages.

#### 3.2.4 Reduction of CARD9 inhibits phosphorylation of NF‐κB and P38 in peritoneal macrophages in vivo and in vitro

**Figure 5:**
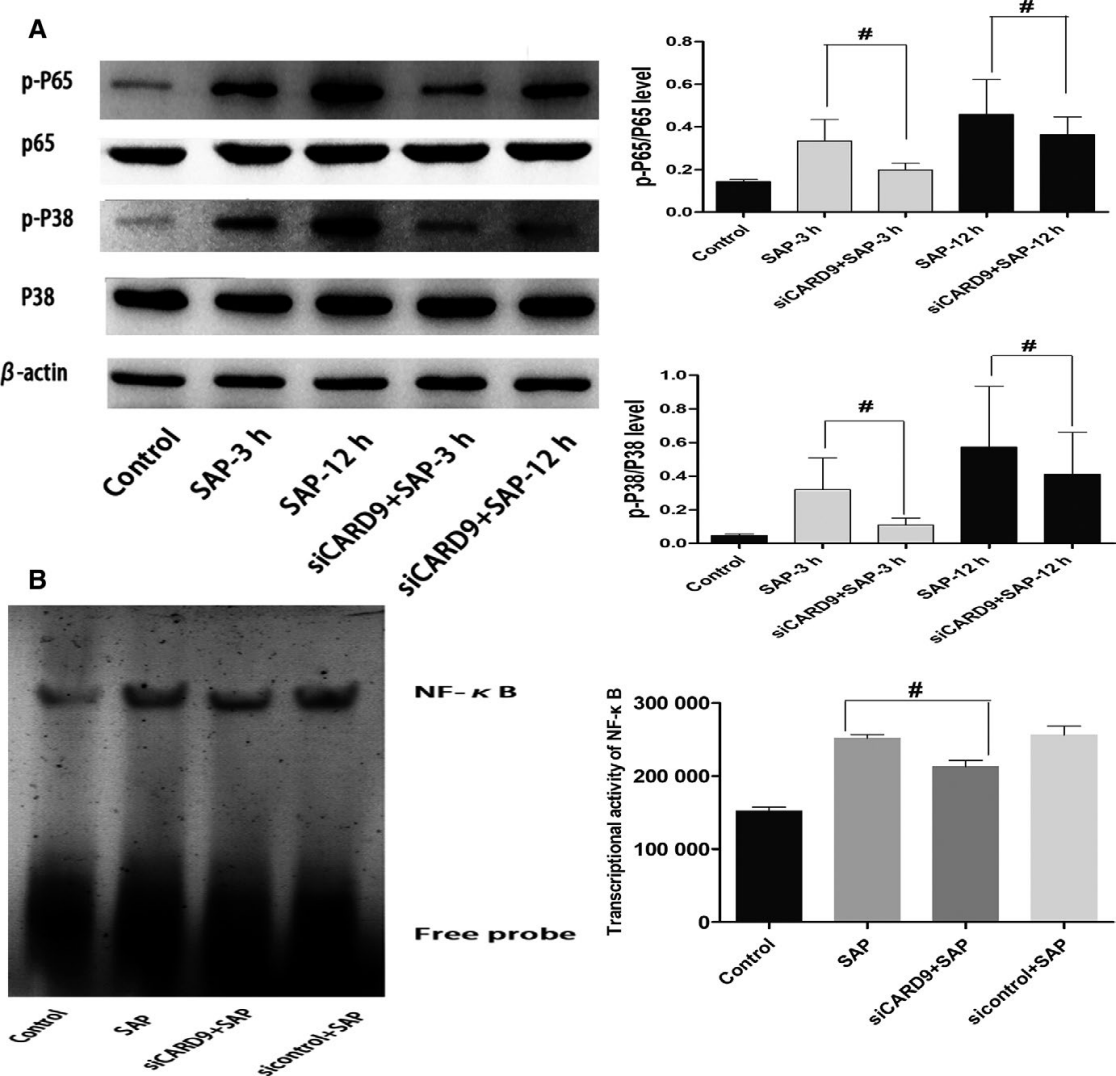
In vivo CARD9 siRNA alleviates Inflammatory reactions in peritoneal macrophages. A, Phosphorylation of NF‐κB and P38 in peritoneal macrophages from each group at the 3‐h and 12‐h time points. The molecular weight of the NF‐κBp65 and P38 bands were 65 kD and 43 kD. B, Transcriptional activity of NF‐κB in peritoneal macrophages from each group. #: SAP group vs siCARD9 group, *P* < .05

To investigate whether CARD9 can regulate the signalling pathways of NF‐κB and p38MAPK in peritoneal macrophages, we applied siRNA interference technology in vivo and in vitro. In vivo, as shown in Figure 5A, the phosphorylation of NF‐κB and P38 in peritoneal macrophages was increased in SAP rats compared with control rats, but decreased in (siCARD9 + SAP) group compared with SAP group, which was consistent with the expression of CARD9 (*P* < .05). In vitro, as shown in Figure 4D, the phosphorylation of NF‐κB and P38MAPK in peritoneal macrophages of each group also changed in coordination with cytokine levels. The phosphorylation of NF‐κB and P38MAPK in peritoneal macrophages in the siCARD9 group was significantly lower than in the SAP and sicontrol groups (*P* < .05). Thus, the data demonstrated that CARD9 acts as a activator in inducing phosphorylation of NF‐κB and P38MAPK in peritoneal macrophages in SAP.

#### 3.2.5 Reduction of CARD9 inhibits transcriptional activity of NF‐κB in peritoneal macrophages in vivo

To explore the effect of CARD9 on the transcriptional activity of NF‐κB, we applied EMSA technology. As shown in Figure 3B, the transcriptional activity of NF‐κB in peritoneal macrophages of SAP rats was increased compared with control rats, which was consistent with the phosphorylation of NF‐κB. The transcriptional activity of NF‐κB in peritoneal macrophages significantly decreased in (siCARD9 + SAP) group compared with SAP group (*P* < .05). Therefore, These results were interpreted to indicate that CARD9 regulates the transcriptional activity of NF‐κB in peritoneal macrophages in SAP.

### 3.3 Reduction of CARD9 reduces the concentration of CARD9 and Bcl10 in peritoneal macrophages of SAP rats

**Figure 6:**
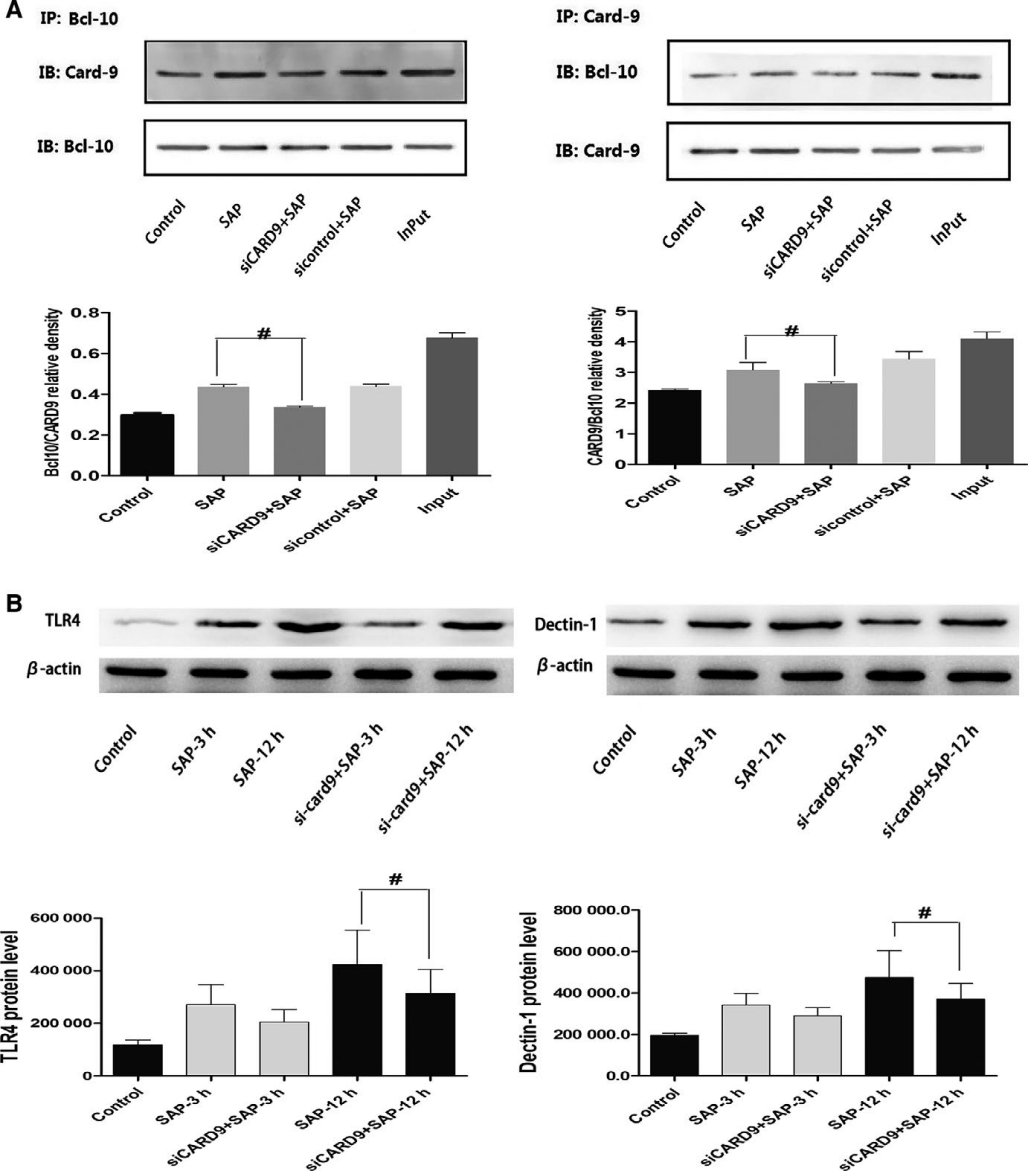
In vivo CARD9 siRNA reduces the concentration of CARD9 and Bcl10 and influences the expression of TLR4/Dectin‐1 in peritoneal macrophages of SAP rats. A, The combination of CARD9 and Bcl10 in peritoneal macrophages from each group. B, The expression of TLR4 and Dectin1 in peritoneal macrophages from each group. The molecular weight of the TLR4 and Dectin1 bands were 95 kD and 28 kD. *: SAP group or siCARD9 or sicontrol group vs control group, *P* < .05; #: SAP group vs siCARD9 group, *P* < .05

It has been reported that CARD9 binds to two proteins containing the CARD domain in macrophages, B‐cell lymphoma factor 10 (Bcl10) and mucosa‐associated tissue lymphoma translocation protein (MALT1), forming a CARD9/BCL10/MALT1 complex (CBM complex). CARD9 activates inflammatory signalling pathways, such as NF‐κB and MAPK in the form of a CBM complex to participate in fungal, bacterial and viral reactions.^5^ To determine if an interaction between CARD9 and Bcl10 occurred in peritoneal macrophages of SAP rats, we utilized immunoprecipitation assays. As shown in Figure 6A, the interaction of CARD9 and Bcl10 could be detected in the control group, SAP group, sicontrol group and siCard9 group. The formation of the complex was greater in the SAP group and sicontrol group, lower in siCard9 group, which was consistent with the phosphorylation of NF‐κB and P38 and transcriptional activity of NF‐κB. These data clearly revealed that Card9 acts on downstream signalling by forming a CARD9/Bcl10 complex in peritoneal macrophages of SAP rats.

### 3.4 The presence of TLR4/Dectin‐1‐CARD9‐NF‐κB/p38MAPK signalling pathway in peritoneal macrophages in SAP

#### 3.4.1 The expression of TLR4 and Dectin1 increased in peritoneal macrophages of SAP rats

Several lines of evidence have been published showing that TLR4 and Dectin‐1 are involved in CARD9 signal transduction in fungal diseases. In order to find the upstream signal of CARD9 in peritoneal macrophages of SAP rats, we detected the expression of TLR4 and Dectin‐1. As shown in Figure 6B, protein expression of TLR4 and Dectin‐1 in peritoneal macrophages of SAP rats was significantly greater than in the control group at 3 hours and remained high at 12 hours (*P* < .05). These data suggested that TLR4 and Dectin‐1 are involved in peritoneal macrophage inflammation in SAP.

#### 3.4.2 TLR4 and Dectin‐1 pathways in macrophages were activated by stimulation with polyinosine and β‐glucan, respectively

**Figure 7:**
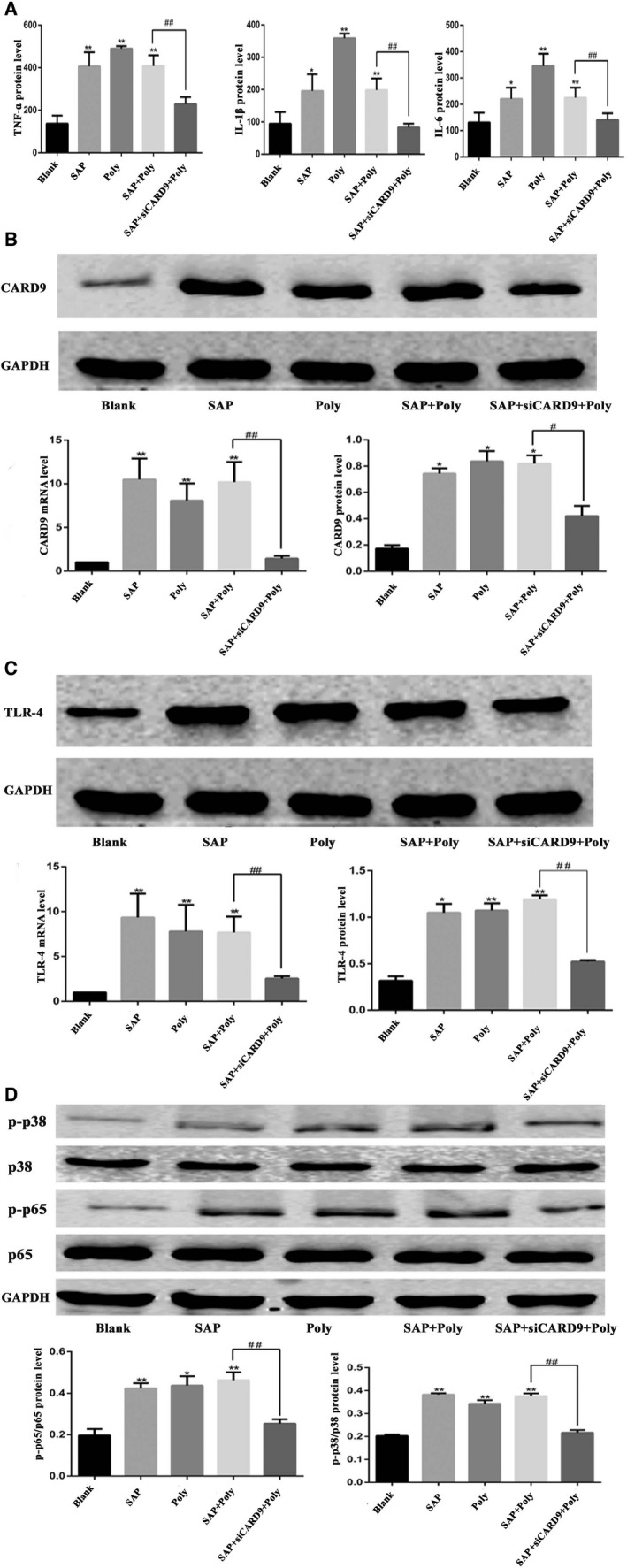
Activation of the TLR4 pathway by polyinosine and suppression of Card9 expression by the si‐Card9 adenovirus. A, The level of TNF‐α, IL‐1βand IL‐6 in cell culture medium from each group. B, mRNA and protein expression of CARD9 in each group. The molecular weight of the CARD9 bands was 62 kD. C, mRNA and protein expression of TLR4 in each group. The molecular weight of the TLR4 bands was 95 kD. D, Phosphorylation of NF‐κB and P38 in peritoneal macrophages from each group at the 6‐h time point. The molecular weight of the NF‐κBp65 and P38 bands were 65kD and 43 kD.*: SAP group or Poly or SAP + Poly group vs Blank group, *P* < .05; **: SAP group or Poly or SAP + Poly group vs Blank group, *P* < .01; #: SAP + siCARD9+ Poly group vs SAP + Poly group, *P* < .05; ##: SAP + siCARD9+ Poly group vs SAP + Poly group, *P* < .01

In order to clarify the relationship between TLR4 and CARD9 in SAP peritoneal macrophages, we used polyinosine to specifically activate the TLR4 pathway of normal peritoneal macrophages and peritoneal macrophages treated with the siCard9 adenovirus. As shown in Figure 7, compared with the blank group, the expression of TLR4 was enhanced after polyinosine treatment. Meanwhile, the levels of TNF‐α, IL‐1β and IL‐6, the phosphorylation of NF‐κB and p38MAPK, and the expression of CARD9 were also increased (*P* < .05). the expression of TNF‐α, IL‐1β, IL‐6 and the phosphorylation of NF‐κB and p38MAPK in SAP rat peritoneal macrophages treated with the siCard9 adenovirus in vitro was lower than that in the SAP group and Poly group (*P* < .05).These data suggested that TLR4 is the upstream of CARD9 and could regulate the expression of CARD9, there may exist a TLR4‐CARD9‐NF‐κB/p38MAPK signalling pathway in peritoneal macrophages in SAP. In addition, we found that the expression of TLR4 was lower in the siCARD9 group in vitro, consistent with the results of in vivo (Figure 6B). We speculated that CARD9 could feedback regulate the expression of TLR4, and there may be exit a TLR4/‐CARD9‐NF‐κB/p38MAPK signalling network in peritoneal macrophages in SAP. We then used β‐glucan to specifically activate the Dectin‐1 pathway in normal peritoneal macrophages and peritoneal macrophages treated with the siCard9 adenovirus. The results showed that the changes were the same as those observed after polyinosine stimulation (Figure S1).

## 4 DISCUSSION

Our study detected a significant increase in the expression of CARD9 in peritoneal macrophages of SAP rats. The expression of CARD9 was consistent with changes in inflammatory response and pro‐inflammatory cytokine expression in peritoneal macrophages, and inhibit the expression of CARD9 could reduce the level of inflammation, suggesting that CARD9 in peritoneal macrophages may have a marked effect on the cascade amplification of inflammatory factors in SAP. Other studies have also reported that CARD9 is involved in inflammatory response, such as inhibit CARD9 signalling could protect the heart from injury.^14^

It has been confirmed that NF‐κB and p38MAPK are classical signalling pathways in SAP, which regulate the production of pro‐inflammatory cytokines.^15^, ^16^ NF‐κB promotes the spread of local inflammatory lesions of the pancreas to the whole body.^17^ P38MAPK is mainly involved in the inflammatory response and apoptosis of cells under various stress conditions.^18^ In our study, the phosphorylation of NF‐κB p65 and p38 MAPK and the transcriptional activity of NF‐κB were attenuated by silencing of CARD9 in peritoneal macrophages of SAP rats in vivo or in vitro. At the same time, the release of inflammatory factors (TNF‐α, IL‐1β, IL‐6) decreased. These results suggested that CARD9 participate in early inflammation of pancreatitis by activating NF‐κB and p38MAPK in peritoneal macrophages Meanwhile, co‐immunoprecipitation assays showed that CARD9 could form a CARD9/Bcl10 complex in peritoneal macrophages to activate the inflammation signalling pathways of NF‐κB and p38MAPK, consistent with previous reports on fungal and viral reactions.^19^, ^20^

CARD9 as an adaptor protein integrates the recognition signals of various innate immune receptors in macrophages, but the upstream regulatory factors of CARD9 in SAP are not yet clear. Macrophages as an innate immune cell express such as membrane‐associated Dectin‐1, TLRs, and other pattern recognition receptors (PPRs), which can through adaptor proteins regulate the intracellular signal transmission to play an important role in the innate immunity. It is reported that activation of TLR4 signalling is involved in SAP, causing inflammatory damage of lung, pancreas and other organs.^21^, ^22^ However, the relationship between TLR4 and CARD9 has not been reported in SAP. By detecting TLR4 expression and observing the effect of TLR4 specific agonist on CARD9 of peritoneal macrophages in SAP, we found that TLR4 participates in activation of CARD9 in SAP, perhaps acting through a TLR4‐CARD9‐NF‐κB/p38MAPK pathway.

Dectin‐1 is another PRR in macrophages which contains an immunoreceptor tyrosine‐activated motif (ITAM)‐like domain and plays a crucial role in the recognition of fungi by binding to the beta‐glucan‐like polysaccharides in their cell walls. ITAM is very important in signal transduction and can mediate intracellular signal transduction and induce a variety of cellular responses. Dectin‐1 plays an important role in natural immunity, which can resist fungal pathogens and bacterial (especially Mycobacterium) infection.^23^, ^24^, ^25^ Evidence has shown that CARD9 is mainly involved in activation of the Dectin‐1‐induced NF‐κB signalling pathway for innate anti‐fungal immunity.^26^, ^27^ However, there has been no research reported to our knowledge about the relationship between CARD9 and Dectin‐1 in the early stage of SAP under conditions of aseptic inflammation. In our study, wu found that Dectin‐1 is a participant in the activation of CARD9 in SAP, there may exist a Dectin‐1‐CARD9‐NF‐κB/p38MAPK pathway.

In addition, we also found that the expression of TLR4 and Dectin‐1 decreased after inhibiting CARD9 expression via RNA interference in SAP peritoneal macrophages in vitro, suggesting that the regulation of TLR4 and Dectin‐1 on CARD9 is not unidirectional; rather, peritoneal macrophages may form an inflammation signalling network, which regulates PPRs. CARD9 may play an important role in this signalling network.

## 5 CONCLUSION

Early diagnosis and treatment of SAP patients are essential to reduce mortality and control disease exacerbation. The role of CARD9 in SAP peritoneal macrophages was verified repeatedly by animal and cell experiments. An inflammation signalling pathway comprised of TLR4/Dectin‐1‐CARD9‐NF‐κB/p38MAPK is likely active in peritoneal macrophages, in which CARD9 plays an important role. Blockade of CARD9 expression can effectively alleviate SAP inflammation, and thus, may be an important therapeutic target for SAP treatment.

## CONFLICT OF INTEREST

No conflicts of interest exist.

## AUTHOR CONTRIBUTION

**Jing Wang:** Conceptualization (equal); Data curation (equal); Formal analysis (equal); Investigation (equal); Methodology (equal); Project administration (equal); Resources (equal); Software (equal); Supervision (equal); Validation (supporting); Visualization (supporting); Writing‐original draft (equal); Writing‐review & editing (equal). **Jun Tian:** Data curation (equal); Formal analysis (equal); Methodology (equal); Project administration (equal); Resources (supporting); Software (equal); Writing‐original draft (equal); Writing‐review & editing (equal). **Yanghuan He:** Data curation (equal); Formal analysis (equal); Methodology (equal); Project administration (equal); Writing‐original draft (equal); Writing‐review & editing (equal). **Zhiwen Yang:** Formal analysis (equal); Investigation (equal); Supervision (equal); Validation (equal). **Lin Wang:** Data curation (supporting); Methodology (supporting); Software (supporting); Supervision (supporting). **Yuexing lai:** Methodology (supporting); Software (supporting); Validation (supporting). **Ping Xu:** Conceptualization (lead); Data curation (equal); Formal analysis (lead); Project administration (lead); Resources (lead); Supervision (lead); Writing‐original draft (equal); Writing‐review & editing (lead).

## Supporting information

Figure S1

## Data Availability

All data models are available from the corresponding author by request.
