# Supplementary figures and images for: Role of CARD9 in inflammatory signal pathway of peritoneal macrophages in severe acute pancreatitis
Source: J Cell Mol Med. 2020 Aug 12;24(17):9774–85. doi: 10.1111/jcmm.15559 (PMC7520331; doi:10.1111/jcmm.15559)

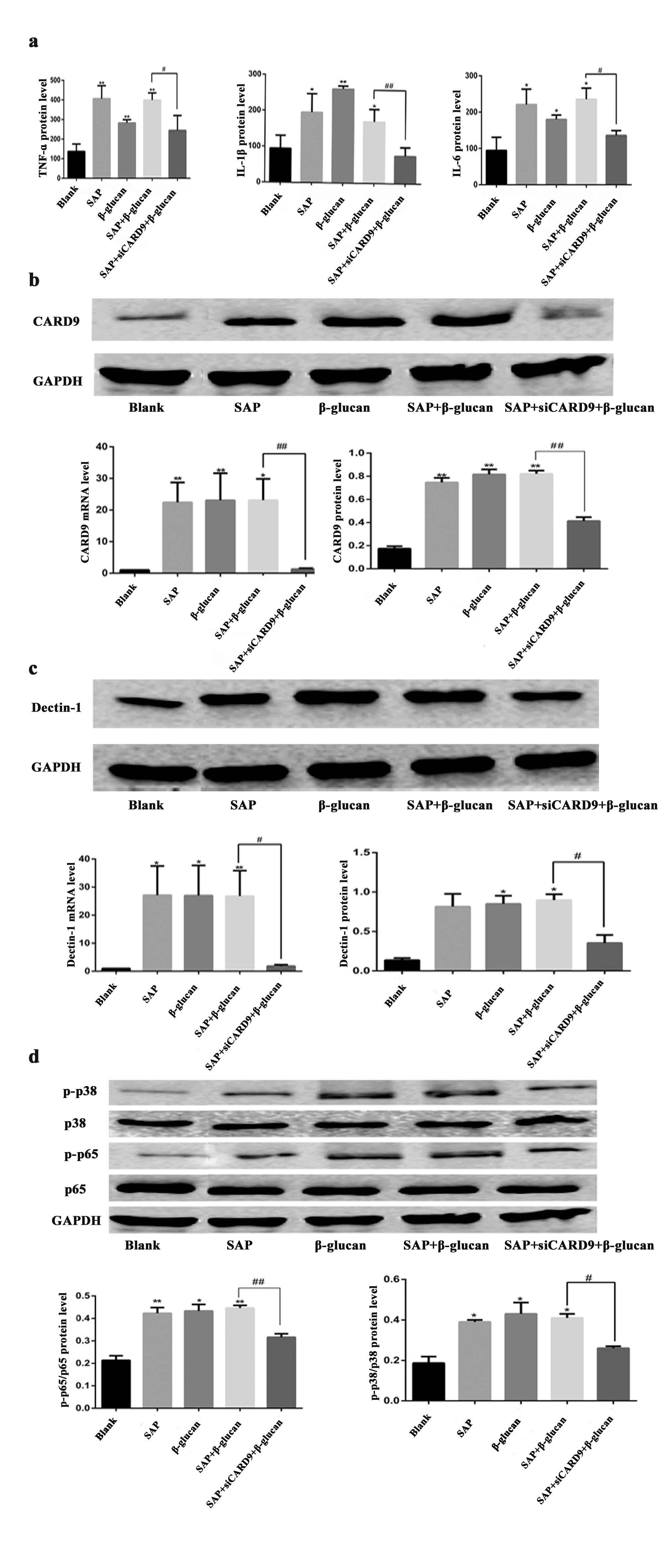

Supplement: Supplementary file 1 — Figure S1 [file JCMM-24-9774-s001.tif]
